# Ring roads and urban biodiversity: distribution of butterflies in urban parks in Beijing city and correlations with other indicator species

**DOI:** 10.1038/s41598-019-43997-8

**Published:** 2019-05-21

**Authors:** Kong-Wah Sing, Jiashan Luo, Wenzhi Wang, Narong Jaturas, Masashi Soga, Xianzhe Yang, Hui Dong, John-James Wilson

**Affiliations:** 1 South China DNA Barcoding Center, Kunming Institute of Zoology, Chinese Academy of Sciences, 650223 Kunming, Yunnan P.R. China; 2 State Key Laboratory of Genetic Resources and Evolution, Kunming Institute of Zoology, Chinese Academy of Sciences, 650223 Kunming, Yunnan P.R. China; 3 Institute of Ecology and Geobotany, School of Ecology and Environmental Science, Yunnan University, Kunming, Yunnan P.R. China; 4 Wildlife Forensic Science Service, Kunming, Yunnan P.R. China; 5 Guizhou Academy of Testing and Analysis, Guiyang, Guizhou P.R. China; 6 Department of Microbiology and Parasitology, Faculty of Medical Science, Naresuan University, 65000 Phitsanulok, Thailand; 7 Graduate School of Agricultural and Life Sciences, The University of Tokyo, 1-1-1, Yayoi, Bunkyo, Tokyo, Japan; 8 International College Beijing, China Agricultural University, Beijing, P. R. China; 9 Fairy Lake Botanical Garden, Shenzhen and Chinese Academy of Sciences, 518004 Shenzhen, Guangdong P.R. China; 10 Vertebrate Zoology at World Museum, National Museums Liverpool, William Brown Street, L3 8EN Liverpool, United Kingdom

**Keywords:** Urban ecology, Entomology

## Abstract

The capital of China, Beijing, has a history of more than 800 years of urbanization, representing a unique site for studies of urban ecology. Urbanization can severely impact butterfly communities, yet there have been no reports of the species richness and distribution of butterflies in urban parks in Beijing. Here, we conducted the first butterfly survey in ten urban parks in Beijing and estimated butterfly species richness. Subsequently, we examined the distribution pattern of butterfly species and analyzed correlations between butterfly species richness with park variables (age, area and distance to city center), and richness of other bioindicator groups (birds and plants). We collected 587 individual butterflies belonging to 31 species from five families; 74% of the species were considered cosmopolitan. The highest butterfly species richness and abundance was recorded at parks located at the edge of city and species richness was significantly positively correlated with distance from city center (*p* < 0.05). No significant correlations were detected between the species richness and park age, park area and other bioindicator groups (*p* > 0.05). Our study provides the first data of butterfly species in urban Beijing, and serves as a baseline for further surveys and conservation efforts.

## Introduction

China is a megadiverse country but is rapidly losing biodiversity as a consequence of socioeconomic development and expansion of urban land since the 1990s^1,2^. Urban land coverage in China expanded 28% from 98,819 km^2^ in 2000 to 126,661 km^2^ in 2010 and is predicted to keep increasing rapidly^3^. With the continuous expansion of urban land across the country, the Chinese government has recognized the key role of urban green spaces, such as urban parks, in the preservation of biodiversity. In order to protect and conserve biodiversity in urban areas, the “Measures for Application and Evaluation of National Garden Cities and the Standards for National Garden Cities” policy^4^ required city developers to build gardens and parks; use native plant species in parks and gardens; and conserve natural landscapes, vegetation, water systems and wetlands as a component of urban planning^4^.

Beijing, the capital city of China, has experienced rapid urban development since the establishment of the People’s Republic of China in 1949, expanding from the inner core (the “Forbidden City”) to retreating outskirts in an elliptical pattern bordered by concentric ring roads^5^. A central business district developed between the 2^nd^ and 3^rd^ ring roads between 1950 and 1980, followed by urban development between the 3^rd^ and 4^th^ ring roads during the next decade (1980s), and then between the 4^th^ and 5^th^ ring road during the following decade (1990s)^5^. Since 2000, former cropland and grassland outside the 5^th^ ring road have been converted into residential and commercial districts^5^; urban land across the wider Beijing Municipality increased from 234 km^2^ to 2720 km^2^ in the last decade^3,6^. The Beijing People’s Government has developed a comprehensive policy for urban greening based on ecological principals, with a long-term goal of connecting urban green spaces, such as parks, with green wedges and corridors to form a sustainable green urban ecosystem^7^.

Insights into the effectiveness of such policies in Beijing, have been provided by surveys of birds^8–10^, plants^2,11–13^, weevils^5^, ground beetles^14^ and insect communities^8^ in urban green spaces, particularly urban parks. Beijing’s parks are important for migratory birds: only 37% of the 52 bird species recorded during the breeding season were resident^10^, suggesting that Beijing is a major node in the East Asian-Australasian Flyway. Beijing’s urban parks have a high number of exotic plant species that were introduced into parks as ornamentals and most rare tree species are only found in older parks^6,15^. In general, urban parks located further from the inner core retain higher species richness of birds and plants^6,8–10,15^. High species richness of breeding birds was recorded from parks that maintained high species richness of insects, probably because insects are the main food source for birds to raise their nestlings^8^. Species diversity of willow trunk-dwelling weevils decreased along a gradient of increasingly intensive urbanization^5^.

Studies of biodiversity in urban parks in other cities suggest that local variables (e.g., size of park) and landscape factors (e.g., proportion of impervious surface) are important determinants of insect and bird diversity^16^. For example, park size was significantly positively correlated with the diversity of butterflies in Shenzhen, China^17^. Positive correlations between park size and butterfly species richness in city may be because larger parks tend to encompass greater habitat diversity^16^, plants species diversity^16^ and microhabitat heterogeneity^17^ than smaller parks – these are each are important factors for survival and reproduction of butterflies^16^.

Surprisingly, to the best of our knowledge, there have been no reports of the distribution and influence of urbanization on butterflies in Beijing. Butterflies, diurnal Lepidoptera, have often been the focus of urban biodiversity studies^18^ (see Ramirez-Restrepo & McGregor-Fors,^18^) because butterflies are thought to react rapidly to environmental changes due to their high mobility and short generation time^19^. Patterns of butterfly diversity are often reflected in other distantly related taxonomic groups^20,21^, including other “indicator species” such as birds and plants. Studies from European cities found the species richness of butterflies and plants is significantly positively correlated^22,23^. A potential warning note from another ancient capital, Rome, is the finding that the highest rates of extirpation of butterflies over Rome’s long history occurred during a period of intense urbanization between 1871 and 1930^21^. In this study, we conducted the first butterfly survey of Beijing’s urban parks with the aim to: (i) estimate the total number of butterfly species in surveyed urban parks; (ii) study the distribution pattern of butterfly species in a megacity; and (iii) examine the correlation between butterfly species richness with park variables (age, area and distance from city center) and species richness of other indicator species (birds and plants).

## Results

(i) **Butterfly diversity in Beijing’s urban parks**
  A total of 587 individual butterflies belonging to 31 species were collected from ten urban parks in Beijing (Table 1). Of the 31 species, twelve species belonged to the family Nymphalidae, nine to Lycaenidae, four to Papilionidae, four to Pieridae and two to Hesperiidae (Table 1). Twenty-one species (68%) were only sampled in a single park (Table 1). Nineteen species (61%) were represented by fewer than five individuals (Table 1). Nearly half (47%) of the collected butterfly individuals were *Pieris rapae* (Table 1). *Pieris rapae* and *Pontia daplidice* were the only species recorded from all ten surveyed parks (Table 1). Of the 31 species recorded, eight species (26%) are endemic to the Sino-Japanese region whereas the other 23 species (74%) are widespread/cosmopolitan (Table 1). The Chao2 estimates of butterfly species richness ranged from 1 to 16 species per park (Table 2). The highest butterfly species richness was observed in Beijing Botanical Garden, which also had the highest estimated species richness based on Chao 2 (16 species, Table 2).

**Table 1:** Butterflies species and abundance recorded in ten urban parks in the Beijing city, June–July 2017. The 10 sampled parks and their abbreviations are: Beijing Botanical Garden (BBG), Chao Yang park (CYP), Jing Shan park (JSP), Liu Yin park (LYP), Nan Hai Zhi park (NHZ), Olympic Forest park (OFP), Tian Tan park (TTP), Yi He Yuan park (YHY), Yuan Ming Yuan park (YMY) and Zhong Shan park (ZSP).

Species	Distribution	BBG	CYP	JSP	LYP	NHZ	OFP	TTP	YHY	YMY	ZSP
**Papilionidae**											
*Papilio maackii*	Endemic	1	0	0	0	0	0	0	0	0	0
*Papilio machaon*	Widespread	0	0	0	0	2	0	0	0	0	0
*Papilio xuthus*	Widespread	1	0	4	1	1	0	2	1	1	0
*Sericinus montela*	Endemic	9	0	0	0	0	0	0	0	0	0
**Pieridae**											
*Colias erate*	Widespread	0	0	0	0	10	5	7	0	1	0
*Pieris canidia*	Widespread	13	0	0	0	0	0	0	1	0	0
*Pieris rapae*	Widespread	19	23	31	45	14	41	47	7	33	15
*Pontia daplidice*	Widespread	11	8	4	1	21	4	26	2	12	1
**Nymphalidae**											
*Apatura ilia*	Widespread	0	0	0	0	2	0	0	2	2	0
*Argynnis hyperbius*	Widespread	1	0	0	0	0	0	0	0	0	0
*Argynnis laodice*	Widespread	3	0	0	0	0	0	0	0	0	0
*Danaus chrysippus*	Widespread	0	0	1	0	0	0	0	0	0	0
*Fabriciana nerippe*	Endemic	0	0	0	0	0	1	0	0	0	0
*Hestina persimilis*	Widespread	1	0	0	0	0	0	0	0	0	0
*Melitaea didymoides*	Endemic	0	1	0	0	0	0	0	0	0	0
*Neptis sappho*	Widespread	3	0	0	0	0	0	0	0	0	0
*Polygonia c-album*	Widespread	1	0	0	0	0	0	0	0	0	0
*Tirumala limniace*	Widespread	0	0	0	0	0	1	0	0	0	0
*Vanessa cardui*	Widespread	0	1	1	0	1	0	0	0	0	0
*Ypthima motschulskyi*	Endemic	3	0	0	0	0	0	0	0	0	0
**Lycaenidae**											
*Celastrina argiolus*	Widespread	0	0	0	0	0	0	1	0	0	0
*Cupido argiades*	Widespread	0	3	0	2	4	21	0	0	0	0
*Lycaena dispar*	Widespread	0	0	0	0	1	0	0	0	0	0
*Lycaena phlaeas*	Widespread	3	0	0	0	0	0	0	0	0	0
*Plebejus idas*	Widespread	0	0	0	0	6	4	0	0	0	0
*Rapala caerulea*	Endemic	1	0	0	0	0	0	0	0	0	0
*Rapala rectivitta*	Endemic	15	0	0	0	0	0	0	0	0	0
*Satyrium w-album*	Widespread	0	0	0	0	0	1	0	0	0	0
*Tongeia filicaudis*	Endemic	3	0	18	0	15	6	4	13	2	0
**Hesperiidae**											
*Lobocla bifasciata*	Widespread	1	0	0	0	0	0	0	0	0	0
*Ochlodes subhyalina*	Widespread	13	0	0	0	0	0	0	0	0	0

**Table 2:** General and historical characteristic and the total observed and Chao 2 estimated butterfly species richness (95% confidence interval) in ten urban parks, Beijing, China.

Urban zone (UZ)	Park	Established (Year)	Area (ha)	Distance from city center (km)	Park functional features	Observed	Chao 2 (95% confidence interval)
1	Zhong Shan park (ZSP)^a,b^	1914	30	0.8	Specific architecture-dominant, old historic temple sites	2	1 (1–2)
1	Tian Tan park (TTP)^a,b^	1420	273	11.7	Specific architecture-dominant, old historic temple site	6	8 (7–20)
1	Jing Shan park (JSP)^a,b^	1179	25	0.9	Old park on composite green space and old architecture	6	5 (4–12)
2	Liu Yin park (LYP)^b^	1984	17	4.8	Green space-dominant with high number of willow trees	4	7 (4–32)
3	Chao Yang park (CYP)^a,b^	1984	355	7.8	Green space- and modern architecture-dominant	5	7 (5–18)
4	Yi He Yuan park (YHY)^b^	1765	290	13.5	Green space-dominant, old imperial park	6	9 (6–26)
4	Yuan Ming Yuan park (YMY)^a,b^	1709	408	5.0	Green space-dominant, old imperial garden	6	9 (6–26)
4	Olympic Forest park (OFP)^a^	2003	680	13.0	Green space-dominant	9	5 (4–12)
5	Nan Hai Zhi park (NHZ)^a^	2009	337	10.6	Green space-dominant	11	10 (9–18)
5	Beijing Botanical Garden (BBG)	1955	400	11.4	Green space-dominant, botanical garden	18	16 (14–26)

^a^Park where species richness of birds had been surveyed by Morelli *et al*.^9^.

^b^Park where species richness of plants had been surveyed by Li *et al*.^15^.
(ii) **Distribution pattern of butterfly species in the Beijing city**
  The butterfly species richness recorded in urban parks ranged from two species (in UZ1) to 18 species (in UZ5) (Fig. 1). The highest butterfly species richness was recorded in Beijing Botanical Garden (UZ5; 18 species). The park closest to the Forbidden City, Zhong Shan Park, had the lowest species richness, with only two species sampled (UZ1; Fig. 1). Seven out of the eight butterfly species (Table 1) recorded in this study that are endemic to the Sino-Japanese region were found in Beijing Botanical Garden located in UZ5.

**Figure 1:**
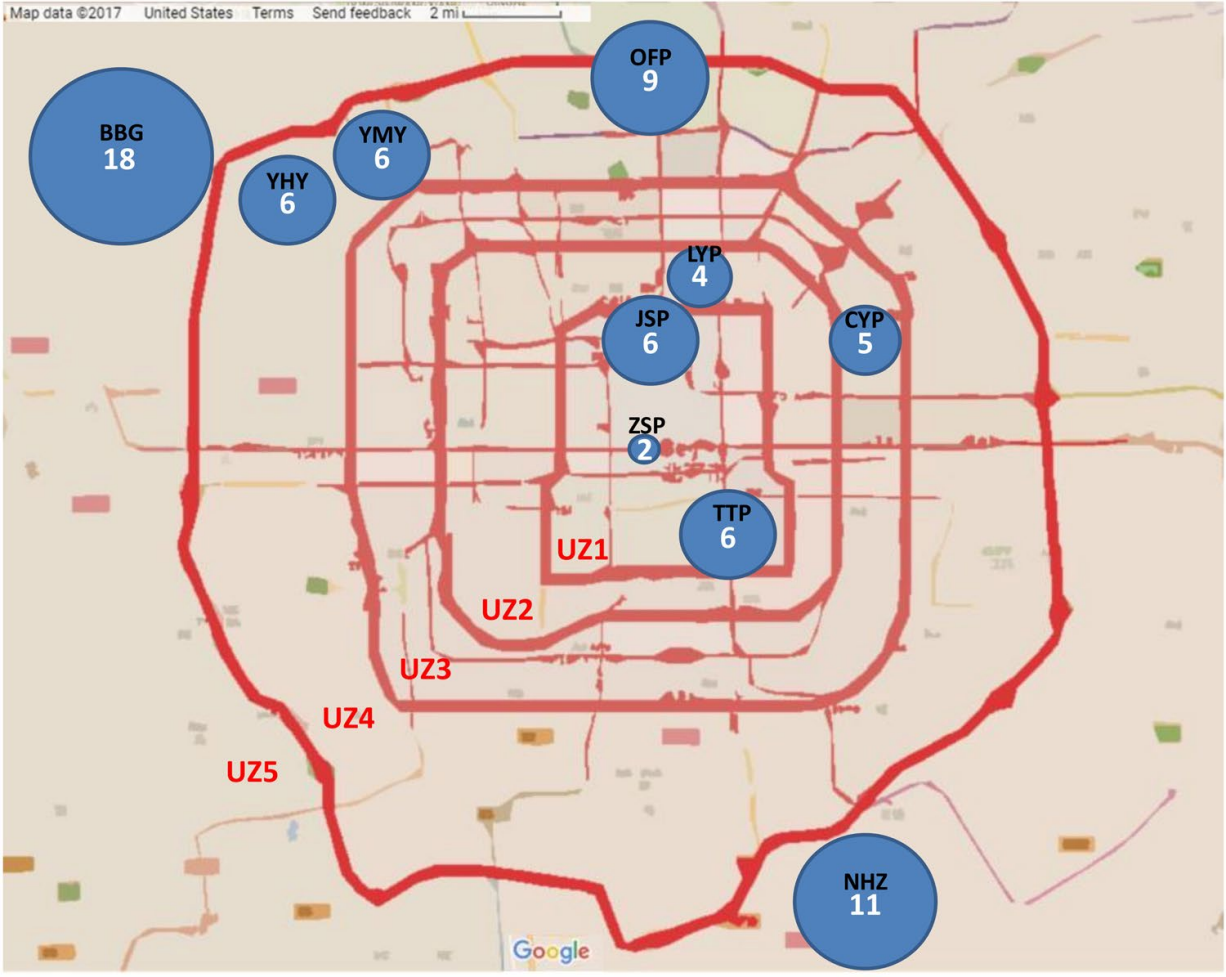
Number of butterfly species recorded in ten urban parks in Beijing city, China, during surveys in June–July 2017. The 10 sampled parks and their abbreviation are: Beijing Botanical Garden (BBG), Chao Yang park (CYP), Jing Shan park (JSP), Liu Yin park (LYP), Nan Hai Zhi park (NHZ), Olympic Forest park (OFP), Tian Tan park (TTP), Yi He Yuan park (YHY), Yuan Ming Yuan park (YMY), Zhong Shan park (ZSP).
(iii) **Correlation of butterfly species richness with park variables** (**age**, **area and distance from city center**) **and other indicator species** (**birds and plants**) **in urban parks**
  The correlations between species richness and park age (*p* = 0.821; Fig. 2), and area (*p* = 0.159; Fig. 2), were not statistically significant (at *p* < 0.05). The correlation between species richness and distance from city center (*p* = 0.048; Fig. 2) was statistically significant (at *p* < 0.05).

**Figure 2:**
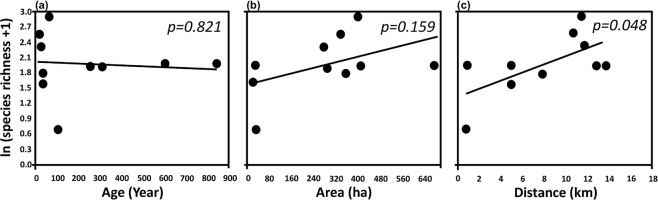
Scatterplots of observed butterfly species richness and (**a**) park age, (**b**) park area, and (**c**) distance from city center in Beijing, China. Surveys were conducted in June–July 2017.

**Figure 3:**
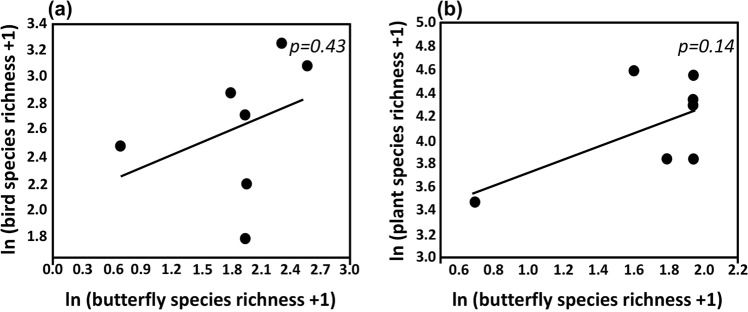
Scatterplots of observed (**a**) bird species richness (n = 7) from Morelli *et al*.^9^ and (**b**) plant species richness (n = 7) from Li *et al*.^15^ against butterfly species richness in urban parks, Beijing, China^61,62^.

Seven of the urban parks we surveyed for butterflies were also surveyed for birds by Morelli and colleagues^9^. Likewise, seven parks had also been surveyed for plants by Li and colleagues^15^. The correlations between butterfly species richness and bird species richness (*p* = 0.43) and butterfly species richness and plant species richness (*p* = 0.14) were both positive, but not statistically significant (at *p * < 0.05; Fig. 3).

## Discussion

There are more than 70,000 cities and towns in China of which 16 are defined as megacities with a population exceeding 5 million^24^. Ramirez-Restrepo and colleagues^25^ estimated that more than one million butterflies inhabit Xalapa, Mexico, a city with a human population of half a million. Such data about butterfly populations are useful for urban management and planning, as well as environmental education^25^. A comprehensive review of urban butterfly studies found only one of 173 studies published between 1956 and 2015 was from mainland China^18^. This was the report of our butterfly survey in Shenzhen^17^. There are additional data available in Chinese language publications, but as far as we know, the present study is the first report of the species richness and distribution of butterflies in Beijing, the Chinese capital.

Five hundred and eighty-seven butterflies representing 31 species from five families were sampled across ten urban parks in the city of Beijing, representing 2.5% of the known butterfly fauna of China^26^. Although our sampling period was limited, the number of butterfly species collected in 60% of the surveyed parks approached asymptote and the rest was similar (different by one to three species) to the predicted species richness, suggesting the sampling effort was sufficient to address our objectives. Nevertheless, the short sampling period may have meant we missed butterfly species with short flying seasons i.e. which only appear in late summer, or inter-annual differences that may be uncovered through further studies.

The total species count of this study is similar to studies from other megacities in the Sino-Japanese and East Palearctic zoogeographic region: 31 butterfly species were recorded in four urban parks in Seoul, South Korea^27^, and 30 butterfly species in Osaka City, Japan, a city that experienced prolonged urbanization between the early 1930s and late 1980s^28^. However, the total species count is lower than those reported from other megacities in China. Forty-three butterfly species were recorded in urban green spaces in Guangzhou^29^; 73 species in 10 urban parks in Shenzhen^18^ and 58 species in 13 parks in Hong Kong^30^. All three cities are located in the Pearl River Delta in subtropical southern China, close to the boundary of the Sino-Japanese and East Palearctic and Oriental zoogeographic regions^31^, an area of higher butterfly diversity compared to northern China^26^. Another possible factor contributing to the relatively low butterfly species richness in Beijing’s urban parks is the lack of an intrinsic ecological concept in the design of most surveyed parks^15^. Half of the surveyed parks in this study were imperial parks/gardens which might not yet fully apply the recently introduced green policies and tend to be dominated by architectural landscapes, including historic edifices, with the main emphasis of park design on aesthetics and recreation^15^.

Twenty-four butterfly species recorded in Beijing’s parks have wide geographic ranges. For example, fourteen of these species were also reported from urban parks in Seoul, South Korea^27^. These species likely persist in urban parks because they can exploit a wide range of food resources^27^ and have good dispersal abilities which allow them to occupy a broad range of ecological niches^27,32^. *Pieris rapae* was the most common and abundant butterfly species collected in Beijing parks. This is similar to the findings from both Chicago, United States of America^33^ and Seoul^27^, where *P*. *rapae* was the most common species in these cities. The success of *P*. *rapae* in urban habitats is attributed to the ability of the butterfly to: disperse^34^, utilize a variety of plants as food sources as both adult and larvae^35^, move through and locate floral resources in heavily developed landscapes^33^, tolerate high temperatures, and use ephemeral habitats within urban green spaces^36^.

The *Danaus chrysippus*, recorded at Jing Shan Park in this study, represents the first record of the species in northern China. The species previously has only been reported in southern provinces, the most northern being Shaanxi^26^, which is ~500 km (shortest distance) from Beijing (Supplemental Fig. 1). Burton^37^ collated range distribution data for *D*. *chrysippus* over the past two decades and revealed that the range had increased considerably and extended north from North African coastal regions to the southern coastal regions of Europe. Likewise, studies in Europe have suggested 63–75% of European Lepidoptera have extended their ranges northward in response to environmental changes such as climatic and/or habitat alteration^37,38^. However, releasing butterflies at special occasions^39,40^ such as weddings, and such an event might explain the presence of *Danaus chrysippus* and another tiger butterfly, *Tirumala limniace*, in Beijing. Both adult butterflies and larvae host plants have only previously been reported in southern China. Further records and surveys of larval host plants are required to verify whether these species have expanded their range northward in China, as only a single individual butterfly of each species was collected in our study.

The butterfly species richness in Beijing parks showed a significant positive relationship with distance from city center. This result was consistent with other studies that found park isolation overrides other park variables (e.g. park area) as a predictor of butterfly species richness^41–43^, with a pattern of decreasing species richness in parks along a rural-urban gradient. Butterfly communities in urban parks are strongly influenced by the surrounding landscape matrix that may act as an environmental filter excluding butterfly species, particularly those with narrow ecological niches^41,42^.

In the present study, the highest butterfly species richness and abundance was recorded from the two parks in UZ5. Notably, 18 species were recorded at Beijing Botanical Garden, a park that adjoins a large area of semi-natural landscape suggesting that preservation of natural landscapes adjoining the city will likely be crucial for effective urban butterfly conservation^43^. This is consistent with findings from Singapore^43^ and Shenzhen^17^ where urban parks adjoining forest retained higher butterfly species richness compared to isolated urban parks that had small areas or impoverished floras^43^. As expected, parks in the inner core had very low species richness. Tian Tan Park (known in English as Temple of Heaven), an ancient park with larger green spaces compared to Zhong Shan Park in UZ1 had a higher species richness (six butterfly species versus two butterfly species).

No significant correlations linking butterfly species richness with park age and park area were found for our surveyed parks. The butterfly species richness in Beijing parks showed positive relationships with the species richness of birds and plants, but the correlations were weak and not statistically significant. Plants, butterflies, and birds are often directly connected in food webs; leaves are the food resources for butterfly larvae and butterfly larvae are food resources for birds^8^. Significant positive correlations between the species richness of butterflies and plants in urban spaces have been observed in the city of Halle/Saale, Germany^22^ and Sheffield, United Kingdom^23^. However, the patterns observed in single taxon such as the butterflies is unlikely to broadly represent how other taxa might be distributed in a heterogeneous urban setting^22^. Further surveys in other urban green spaces such as university gardens and the outlying suburbs of Beijing city may be necessary to reveal any correlation between butterfly species richness and the examined variables in this study as the number of public parks providing permission to sample butterflies was limited.

In urban areas, butterflies provide important ecosystem services and are an ideal animal group through which to reconnect people with nature^44^. Similarly to other studies, most of the butterflies in Beijing urban parks are common species. Globally, an increasing number of common species of birds, fish and insects (including some butterflies) are in decline and require suitable monitoring to support urban wildlife policy and management^45,46^. Between 1992–2007, butterfly species that used to be omnipresent in gardens and parks in the Netherlands suffered severe declines under land-use change pressure^46^.

In the absence of historical records of butterfly diversity in Beijing, our findings serve as baseline data for further surveys. Data on the distribution and species richness of butterflies in urban landscapes are particularly valuable for the development of butterfly conservation strategies but are currently lacking in China. An experimental butterfly conservation project in Beijing city organized by the Shan Shui Conservation Center has the aim of enhancing the richness of native butterfly species in Beijing by planting host plants in residential areas^47^. Management schemes and techniques for conserving butterflies in urban parks in megacities like Beijing, that regularly receive large number of visitors or are dominated by structures with historical value, are especially valuable. Habitat-specific management strategies such as setting unmanaged areas in urban parks^15,17^ and linking isolated urban parks through greenways could be effective in improving the ability of parks to sustain butterfly populations in the growing number of Asian megacities.

## Conclusion

The total species count of this study is similar to studies from other megacities in the Sino-Japanese and East Palearctic zoogeographic region. Nearly three quarters (74% of 31 species) of the total observed species in urban parks in Beijing were considered widespread. Similar to other urban butterfly studies, decreasing butterfly species richness in parks along a rural-urban gradient was observed in Beijing.Parks located at the edges of Beijing city supported the highest butterfly species richness and abundance, suggesting that preservation of natural landscape adjoining the city will likely be necessary for effective urban butterfly conservation. Our study provides the first data of butterfly species richness in Beijing serving as baseline data for further surveys and conservation efforts.

## Methods

### Butterfly sampling

Beijing has a temperate, humid, continental climate with hot summers and cold winters; the average temperature is 12 °C^15^. Annually, the city receives 400–500 mm of rain which mostly falls between June–September^15^. Our survey was conducted between June and July 2017 when the temperature is 13–38 °C and humidity is 13–96%, representing the optimal season for adult butterfly activity.

Prior to conducting our butterfly survey in city parks, applications for permission to conduct a butterfly survey were submitted to respective park management offices. Our survey was limited to the parks for which we received permission from the park management. Ultimately, we conducted butterfly sampling in ten urban parks that are roughly evenly spread throughout Beijing city. These parks are open to the general public and are managed by the People’s Government of Beijing Municipality except Chao Yang Park which is operated by private company (Table 2). Four of the surveyed parks are green space-dominant and the rest are dominated by architectural landscapes, including a historic temple site and imperial parks (Table 2). Each park was sampled over three consecutive days comprising 180 minutes each day. We followed an active search timed survey method used in our butterfly surveys in Kuala Lumpur^48^ and Shenzhen^17^, where butterflies were collected during the 180 minutes survey period within accessible areas. This method allowed a full search of green areas in parks and avoided sampling biases due to differences in size and shape between parks. Due to the homogeneity of the landscape in Beijing parks, we did not designate microhabitat types. Butterflies were collected by an experienced collector using a hand net between 09:00 and 14:00 during calm weather to correspond with the peak flight activity period of adult butterflies. A specimen of *Tirumala limniace* found dead on a roadside in Olympic Forest Park during the butterfly sampling, was retrieved and also included in the analysis.

### Butterfly identification and diversity

In this study, we collected some butterflies with broken wings (for example the *Tirumala limniace*) where morphology identification keys would be of limited use. Although it is generally thought that butterflies are easy to identify, this is not necessarily the case, as seen with the large numbers of mis-identified museum specimens. Butterfly species can be delimited unambiguously based on DNA barcodes. We combined both DNA barcoding and morphological methods for species identification for our study. All collected butterflies were brought back to laboratory. DNA was extracted from a single leg, or 2–3 legs in the case of small lycaenids, of each butterfly using TIANamp extraction kit following the manufacturer’s instructions (Tiangen Biotech, Beijing). DNA barcode fragments of COI mtDNA^49^ were amplified (following standard protocols in Wilson,^50^) using LCO1490/HCO2198 primers^51^ as a first pass and mlCOlintF/HCO2198 primers^52,53^ as a second pass. PCR products were Sanger sequenced by a local company and checked for quality (following standard protocols in Wilson, Sing & Jaturas,^54^). Generated DNA barcodes and associated specimen data were submitted to Barcode of Life Datasystems (BOLD; Ratnasingham & Herbert,^55^) under the project “Urban Butterflies in Beijing Parks” (Project code: BJUP)”. In BOLD, the DNA barcodes were automatically sorted into Barcode Index Numbers (BINs; Ratnasingham & Herbert,^56^). 435 DNA barcodes were assigned Linnaean species names when their sequence grouped into a BIN which included DNA barcodes with Linnaean species names submitted by other BOLD users. 110 DNA barcodes were not assigned to any BIN (due to their short sequenced length or because they contained more than 1% ambiguous bases) but were assigned Linnaean species name on the basis of >98% sequence similarity to named DNA barcodes in BOLD. 42 butterflies that failed to generate DNA barcodes were assigned Linnaean species names based on their wing patterns following a local butterfly reference book^26^.

We followed the zoogeographic regions for butterflies developed by Wu *et al*.^31^ and categorized butterfly species as “endemic” when the species was only reported from one zoogeographic region and “widespread” when the species was reported from more than one zoogeographic region (using distribution records from Savela^57^). The predicted butterfly species richness (Chao 2 rarefactions) based on incidences in each urban park was calculated using EstimateS^58^.

### Butterfly distribution in Beijing megacity

In urban ecology, study sites are generally classified into three categories according to the proportion of impervious surface (PIS): urban (PIS > 50%), suburban (20% < PIS < 50%) and rural (PIS < 20%)^59^. Based on the ring road system and the spatial pattern of PIS, Beijing has been divided into five urban zones (UZs) (Fig. 1)^9^. Our survey included three parks in UZ1, one in UZ2, one in UZ3, three in UZ4 and two in UZ5.

### Correlation of butterfly species with park variables and other indicator species in urban parks

The species richness values of butterflies were natural logarithm ln (species richness +1) transformed prior to further analysis.

We calculated Pearson correlation coefficients using Paleontological Statistical software (PAST)^60^ to test for significant correlations between species richness of butterflies and park age, park area and distance from the city central.

We obtained species richness data from published literature for birds^9^, and plants^15^ recorded in any of the ten urban parks in Beijing where we sampled butterflies (Fig. 1, Supplementary Table 1) and calculated Pearson correlation coefficients between species richness of butterflies and the other taxa (in PAST).

## Supplementary information

Supplementary 1
