# Supplementary material for: Ring roads and urban biodiversity: distribution of butterflies in urban parks in Beijing city and correlations with other indicator species
Source: Sci Rep. 2019 May 21;9:7653. doi: 10.1038/s41598-019-43997-8 (PMC6529450; doi:10.1038/s41598-019-43997-8)

Ring roads and urban biodiversity: distribution of butterflies in urban parks in Beijing city and correlations with other indicator species

Kong-Wah Sing[1,2*], Jiashan Luo[3], Wenzhi Wang[1,2,4,5], Narong Jaturas[6], Masashi Soga[7], Xianzhe Yang[8], Hui Dong[9], John-James Wilson[10,6,8*]

Supplementary Table 1: Number of indicator species recorded in ten surveyed urban parks. The 10 sampled parks and their abbreviation are: Beijing Botanical Garden (BBG), Chao Yang park (CYP), Jing Shan park (JSP), Liu Yin park (LYP), Nan Hai Zhi park (NHZ), Olympic Forest park (OFP), Tian Tan park (TTP), Yi He Yuan park (YHY), Yuan Ming Yuan park (YMY), Zhong Shan park (ZSP)

**Indicator Species**	**BBG**	**CYP**	**JSP**	**LYP**	**NHZ**	**OFP**	**TTP**	**YHY**	**YMY**	**ZSP**
Butterflies	18	5	6	4	12	9	6	6	6	2
Birds	-	17	5	-	21	25	8	-	14	11
Plants	-	46	45	96	-	-	73	94	71	31

Supplementary Figure 1: The distribution of *Danaus chrysippus* and its recent range expansion[61]. The *Danaus chrysippus* photo comes from http://malaysiabutterflies.myspecies.info (J.J.W)[62].

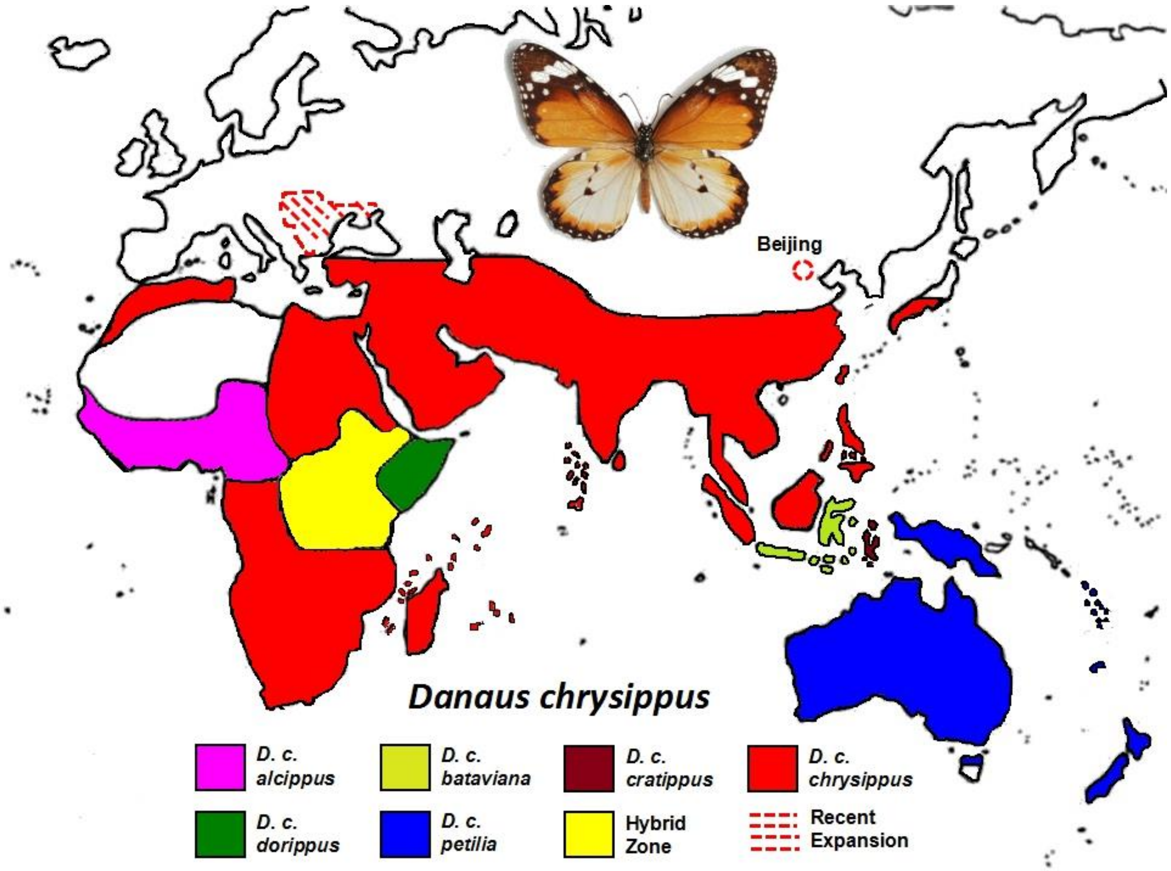

Supplement: Supplementary file 1 — Supplementary 1 [file 41598_2019_43997_MOESM1_ESM.pdf]
